# Correction to “Celastrol Induces Proteasomal Degradation of FANCD2 to Sensitize Lung Cancer Cells to DNA Crosslinking Agents”

**DOI:** 10.1111/cas.70066

**Published:** 2025-03-29

G.‐Z. Wang, Y.‐Q. Liu, X. Cheng and G.‐B. Zhou, “Celastrol Induces Proteasomal Degradation of FANCD2 to Sensitize Lung Cancer Cells to DNA Crosslinking Agents,” *Cancer Science* 106 (2015): 902–908, https://doi.org/10.1111/cas.12679.

In Figure 2b, there were errors in the images of the Actin bands. The correct images for Figure 2b are shown below:

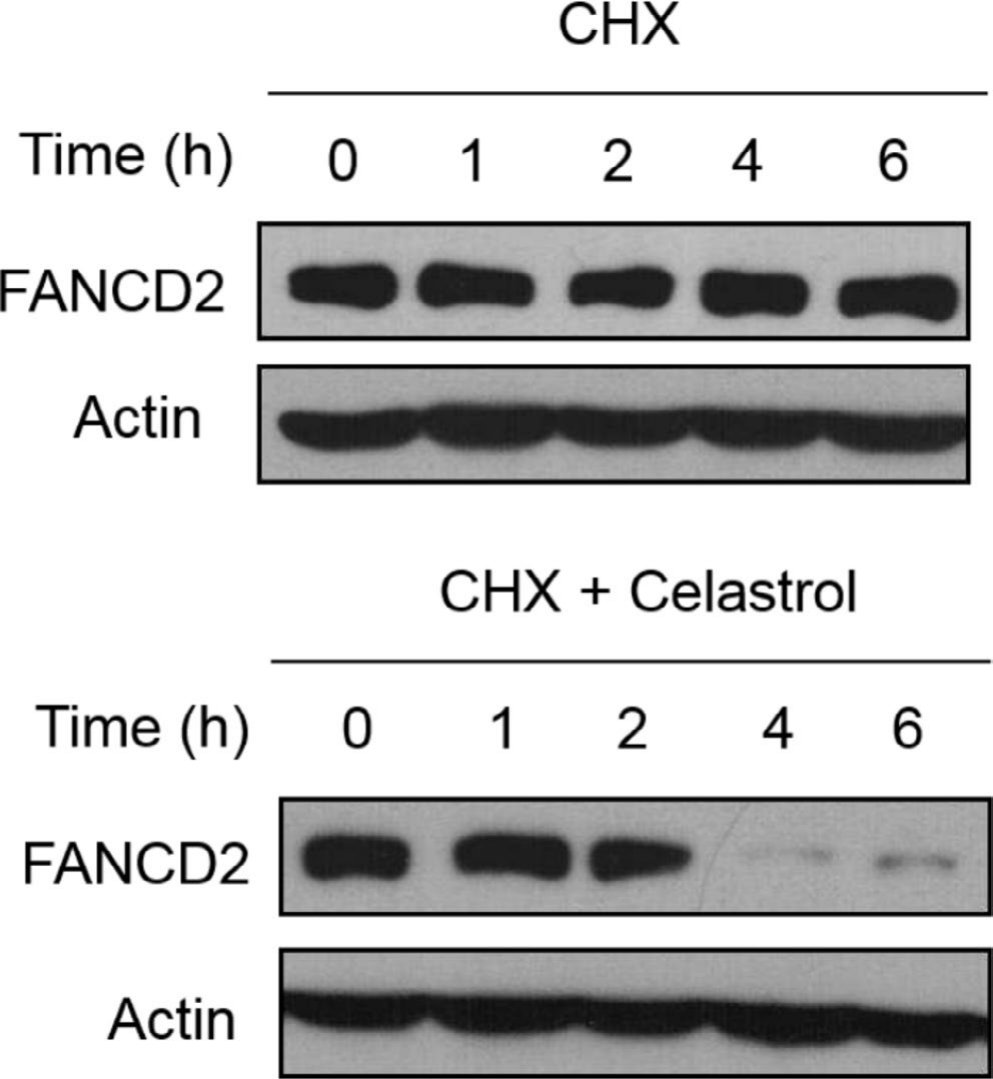

The authors apologize for the error.

